# Borna Disease Virus Phosphoprotein Impairs the Developmental Program Controlling Neurogenesis and Reduces Human GABAergic Neurogenesis

**DOI:** 10.1371/journal.ppat.1004859

**Published:** 2015-04-29

**Authors:** Chloé Scordel, Alexandra Huttin, Marielle Cochet-Bernoin, Marion Szelechowski, Aurélie Poulet, Jennifer Richardson, Alexandra Benchoua, Daniel Gonzalez-Dunia, Marc Eloit, Muriel Coulpier

**Affiliations:** 1 INRA, UMR 1161, Maisons-Alfort, France; 2 ANSES, UMR Virologie, Maisons-Alfort, France; 3 Université Paris-Est, Ecole Nationale Vétérinaire d’Alfort, UMR Virologie, Maisons-Alfort, France; 4 Institut National de la Santé et de la Recherche Médicale, UMR 1043, Toulouse, France; 5 Centre National de la Recherche Scientifique, UMR 5282, Toulouse, France; 6 Université Paul Sabatier, Toulouse 3, Toulouse, France; 7 CECS, I-STEM, AFM, Evry, France; 8 Pasteur Institute, Pathogen Discovery Laboratory, Biology of Infection Unit, INSERM U1117, Paris, France; Freiburg University, GERMANY

## Abstract

It is well established that persistent viral infection may impair cellular function of specialized cells without overt damage. This concept, when applied to neurotropic viruses, may help to understand certain neurologic and neuropsychiatric diseases. Borna disease virus (BDV) is an excellent example of a persistent virus that targets the brain, impairs neural functions without cell lysis, and ultimately results in neurobehavioral disturbances. Recently, we have shown that BDV infects human neural progenitor cells (hNPCs) and impairs neurogenesis, revealing a new mechanism by which BDV may interfere with brain function. Here, we sought to identify the viral proteins and molecular pathways that are involved. Using lentiviral vectors for expression of the *bdv-p* and *bdv-x* viral genes, we demonstrate that the phosphoprotein P, but not the X protein, diminishes human neurogenesis and, more particularly, GABAergic neurogenesis. We further reveal a decrease in pro-neuronal factors known to be involved in neuronal differentiation (*ApoE*, *Noggin*, *TH* and *Scg10/Stathmin2*), demonstrating that cellular dysfunction is associated with impairment of specific components of the molecular program that controls neurogenesis. Our findings thus provide the first evidence that a viral protein impairs GABAergic human neurogenesis, a process that is dysregulated in several neuropsychiatric disorders. They improve our understanding of the mechanisms by which a persistent virus may interfere with brain development and function in the adult.

## Author Summary

When a virus enters the brain, it most often induces inflammation, fever, and brain injury, all signs that are indicative of acute encephalitis. Under certain conditions, however, some neurotropic viruses may cause disease in a subtler manner. The Borna disease virus (BDV) is an excellent example of this second class of viruses, as it impairs neural function without cell lysis and induces neurobehavioral disturbances. Recently, we have shown that BDV infects human neural progenitor cells (hNPCs) and impairs neurogenesis, revealing a new mechanism by which BDV may interfere with brain function. In the present study, we identify that a singled-out BDV protein called P causes similar impairment of human neurogenesis, and further show that it leads to diminution in the genesis of a particular neuronal subtype, the GABAergic neurons. We have also found that the expression of several genes involved in the generation and the maturation of neurons is dysregulated by this viral protein, which strongly suggests their implication in P-induced impairment of GABAergic neurogenesis. This study is the first to demonstrate that a viral protein interferes with human GABAergic neurogenesis, a process that is frequently impaired in neuropsychiatric disorders. It may thus contribute to elucidating the molecular bases of psychiatric disorders.

## Introduction

Upon entrance in the brain, viruses most often induce inflammation, fever, and brain injury, all signs symptomatic of acute encephalitis. A strong immunological response is typically triggered and generally limits viral dissemination and resolves infection. In some cases, however, viruses may not be recognized by the immune system, thus allowing their life-long persistence in the central nervous system (CNS). Continuous viral replication may then interfere with cellular functions, and while not causing overt tissue damage may nevertheless lead to disease. This was first recognized in the early 80s when the lymphocytic choriomeningitis virus (LCMV) was shown to disrupt homeostatic and cognitive functions without obvious tissue injury in its natural murine host [1–4]. Rabies is another well-known virus that disrupts brain function in the absence of cell lysis and causes dramatic alteration in behavior in both animals and humans [5]. Such studies conducted in animal models, together with epidemiological analyses of human neuropsychiatric conditions, have suggested that persistent viral infection may play a role in human mental disorders of unclear etiology [6–8]. Understanding the mechanisms by which persistent viruses impair brain function and how this may be related to neurological and neuropsychiatric diseases has thus become a major challenge in neuro-virology.

Borna disease virus (BDV) is a highly neurotropic virus that persists in the CNS of infected individuals for their entire lifespan. It is an enveloped virus with a non-segmented, negative-sense, single-stranded RNA genome that belongs to the *Bornaviridae* family within the *Mononegavirales* order [9,10]. Its small genome of 8.9 kb encodes 6 proteins, the nucleoprotein (N), phosphoprotein (P), X protein (X), matrix protein (M), glycoprotein (G) and polymerase (L). N, P, X and L form the polymerase complex, the smallest unit necessary for genome replication. Natural BDV infection has been identified in a wide range of vertebrates, including horses, sheep, cattle, dogs, cats, shrews, ostriches and non-human primates [11–16]. Infected hosts develop a wide spectrum of neurological disorders, ranging from immune-mediated disease to behavioral alteration without inflammation. The latter includes deficits in learning and social behavior that are reminiscent of symptoms observed in human psychiatric diseases [17,18]. In humans, evidence supporting the presence of BDV in the brain of a schizophrenic patient has been reported [19] and some epidemiologic studies have supported BDV infection [20]. A possible association between BDV infection and psychiatric diseases has, however, been debated for years [21], with the most recent study showing no evidence of association [22]. This controversy may not be fully resolved until measures to ensure reliability, such as those described by Hornig *et al*., (2012) [22], are adopted by all investigators performing BDV diagnosis. Moreover, since detection of BDV is currently hampered by reason of its sequestration in the brain and its weak immunogenicity, the development of new diagnostic tools would improve association studies in the future. Nevertheless, BDV infection provides an excellent model to study the relationship between persistent viruses and the development of chronic neurological symptoms, with possible consequences for public health.

BDV interference with cellular signaling has been evidenced in neuronal and glial cells. In neurons, BDV has been shown to block neurotrophin-induced signaling, leading to diminished neuritic outgrowth [23] and synaptogenesis [24]. It is also known to block synaptic vesicle recycling in response to stimuli-induced synaptic potentiation [25,26] and to limit through its phosphoprotein the mobility of the GABA receptor, two mechanisms by which it may impair synaptic transmission [27]. As regards glial cells, the selective expression of the viral phosphoprotein P in astrocytes led to neurobehavioral disturbances in transgenic mice [28], suggesting that BDV infection of astrocytes may also contribute to behavioral disorders. Beyond its role in highly specialized cells, we have recently demonstrated that BDV infects human neural progenitor cells (hNPCs) in culture and impairs their capacity to produce neurons, thus, identifying a new mechanism by which BDV may interfere with brain function [29]. Neurogenesis is a process that occurs not just during development but also throughout life, although it is restricted to discrete brain areas in adults. Given that abnormality in the generation of neurons, in pre- and post-natal life, is viewed as characteristic of human mood disorders, such as depression, dementia and psychosis [30–32], our work has opened a new field in BDV research.

The goal of this study was to unravel the molecular mechanisms by which BDV impairs neurogenesis in hNPCs. Using transgenic hNPCs expressing the viral genes encoding the P or X protein, we have identified P as a protein responsible for alteration of human neurogenesis. We then provide evidence that P damages GABAergic neurogenesis and, finally, we show that cellular dysfunction is associated with impairment of specific components of the intrinsic molecular program responsible for neurogenesis. To our knowledge, this is the first demonstration that a viral protein interferes with human GABAergic neurogenesis, a process that is dysregulated in several neuropsychiatric disorders. This is also the first study that addresses and elucidates some of the molecular mechanisms responsible for virally induced alteration of human neurogenesis.

## Results

### Establishment of transgenic hNPCs expressing *bdv-p*, *bdv-x* or *gfp* gene

**Fig 1:**
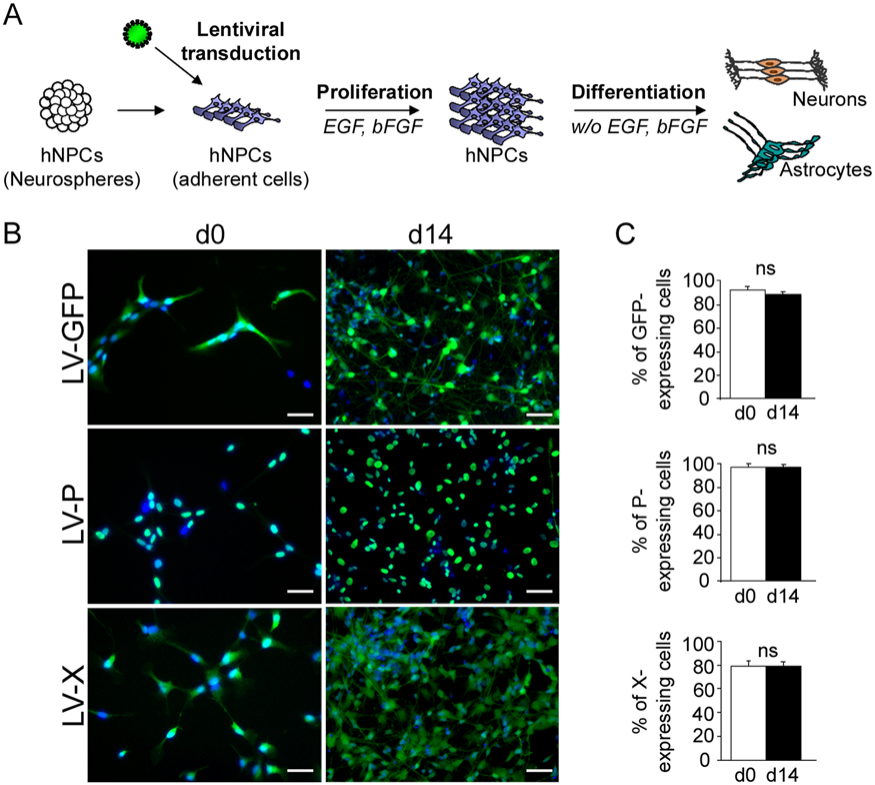
Lentiviral transduction and establishment of transgenic hNPCs. (A) Schematic representation of the experimental procedure. (B) Immunofluorescence labeling of undifferentiated (day 0) and differentiated (day 14) hNPCs, following lentiviral transduction. Antibodies against the viral P (green) or X (green) proteins were used and nuclei were stained with DAPI (blue). Note the localization of the P (nuclear) and the X (nuclear and cytoplasmic) proteins. (C) Evaluation of transduction efficiency based on enumeration of immunostained cells. Results are representative of 3 independent experiments performed in triplicate. Statistical analyses were performed using the Mann-Whitney test. *ns*, non-significant (p > 0.5). Scale bar, 20 μm.

To identify the viral proteins responsible for BDV-induced alteration of neurogenesis, we chose to study the phosphoprotein and the X protein (henceforth referred to P and X), as they have been previously described to interact with many cellular pathways in neural cells. We thus established transgenic populations of hNPCs expressing either *bdv-p* or *bdv-x* gene, or as a control, the *gfp* gene. At 10 weeks of expansion, adherent hNPCs were transduced with highly purified lentiviral vectors encoding the different genes of interest and amplified for a further 2 to 4 week period before epidermal growth factor (EGF) and basic fibroblast growth factor (bFGF) withdrawal and analysis of the effect of the transgene on neural differentiation (Fig 1A). The level of expression of *gfp*, *bdv-p* or *bdv-x* genes in transduced hNPCs was first verified. In undifferentiated cells (day 0) more than 90% of hNPCs were GFP- (91.28 +/- 2.9%) or P-positive (96 +/- 2%) and approximately 80% were X-positive (79 +/- 4.3%), as determined by enumeration of cells labeled with antibodies directed against the P or X viral proteins (Fig 1B and 1C). A similar percentage of transgene-expressing hNPCs was observed after 14 days of differentiation (87.6 +/- 1.1%, 96.2 +/- 1.5%, and 79.9 +/- 3.3% of cells expressing the *gfp*, *bdv-p* or *bdv-x* gene, respectively) (Fig 1B and 1C). Thus, high efficiencies of transduction were obtained with lentiviral vectors and differentiation did not affect *gfp*, *bdv-p* and *bdv-x* gene expression. In keeping with the presence of a nuclear localization signal [33], P was strictly nuclear, whereas X, which contains both a nuclear localization signal and a short helix responsible for mitochondrial targeting [34,35], was observed in both nuclear and cytoplasmic structures, in undifferentiated and differentiated hNPCs (Fig 1B).

### Expression of the *bdv-p* or *bdv-x* gene in hNPCs does not alter their undifferentiated stage and survival

**Fig 2:**
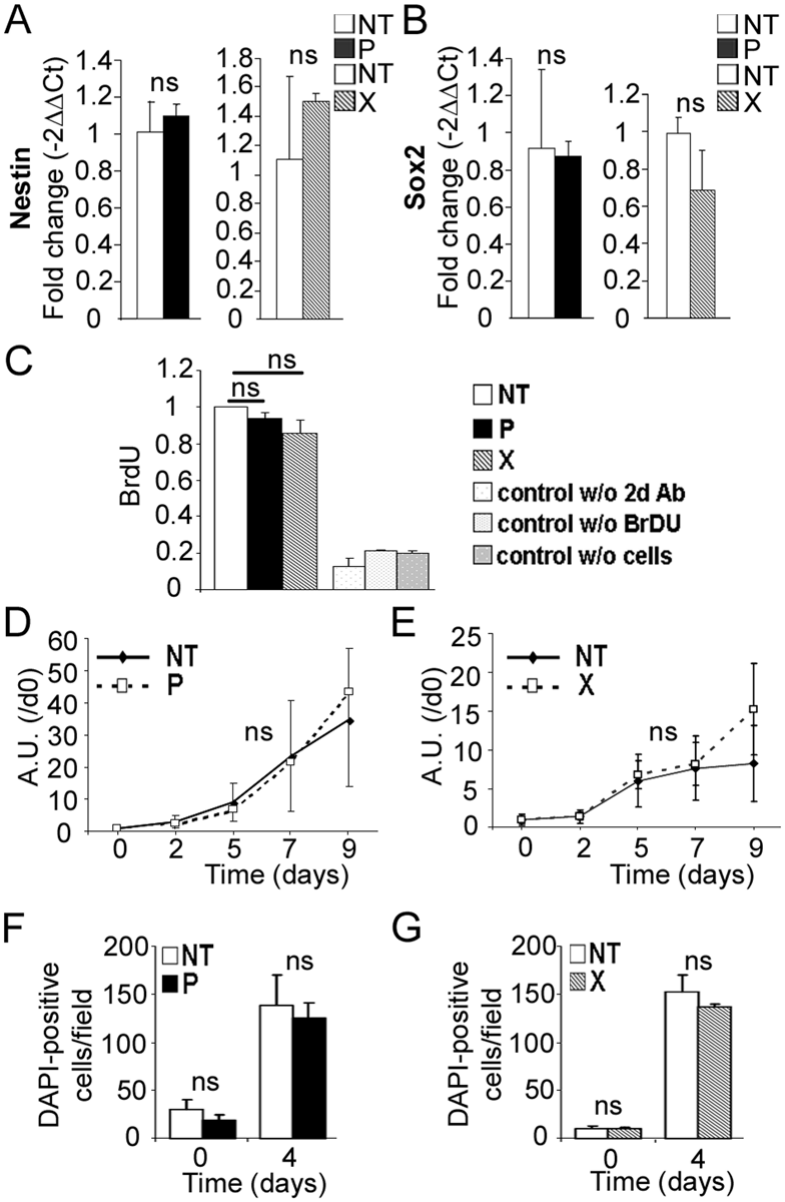
Expression of *bdv-p* or *bdv-x* gene does not alter hNPCs at the undifferentiated stage. RNAs from *bdv-p-* and *bdv-x*-expressing hNPCs and their matched NT controls were analyzed by RT-qPCR for expression of (A) Nestin and (B) Sox2. Proliferation of *bdv-p* and *bdv-x*-expressing hNPCs was analyzed by BrdU labeling (C) and by a mitochondrial dehydrogenase activity-based assay (D and E) in the presence of growth factors and by enumeration of DAPI-positive cells (F and G) in the absence of growth factors. Results in A and B are representative of two independent experiments performed in triplicate. Results in C represent the mean of two independent experiments performed in quintuplicate. Results in D and E are representative of 2 independent experiments performed in quintuplicate. Results in F and G are from 1 experiment performed in triplicate. Statistical analyses were performed using the Mann-Whitney test. *ns*, non-significant (p > 0.5).

At the undifferentiated stage, transgenic hNPCs expressing either the *gfp*, *bdv-p* or *bdv-x* gene were morphologically indistinguishable from their non-transduced (NT) matched controls (S1 Fig). Transgene expression had no impact on cell survival, as observed by light microscopy examination (S1 Fig), and the presence of the viral proteins, P or X, did not modify the expression of Nestin and Sox2, two markers of the undifferentiated stage (Fig 2A and 2B). We next examined whether the viral genes influenced the proliferative capacity of hNPCs. Transgene-expressing hNPCs and their NT matched controls were cultured in the presence of growth factors—EGF and bFGF—and proliferation was determined by evaluation of both BrdU incorporation and mitochondrial dehydrogenase activity. No significant difference was observed between *bdv-p*- and *bdv-x*-expressing hNPCs and their matched NT controls (Fig 2C, 2D and 2E). Since, however, an effect on proliferation may have been masked by the presence of growth factors, we measured proliferation after growth factor withdrawal by enumerating hNPCs at day 0 and day 4 of differentiation using DAPI staining. At day 4, NT hNPCs were 5 to 7.5 fold more numerous than at day 0, showing that cells continued to proliferate in the first days of differentiation. No difference, however, was observed in *bdv-p*- and *bdv-x*-expressing hNPCs compared with their matched NT controls (Fig 2F and 2G), demonstrating that transgenic hNPCs were not impaired in their capacity to proliferate. Thus, the viral P and X proteins did not alter hNPCs at the undifferentiated stage.

### Expression of the *bdv-p* but not *bdv-x* gene reduces the capacity of hNPCs to generate neurons

**Fig 3:**
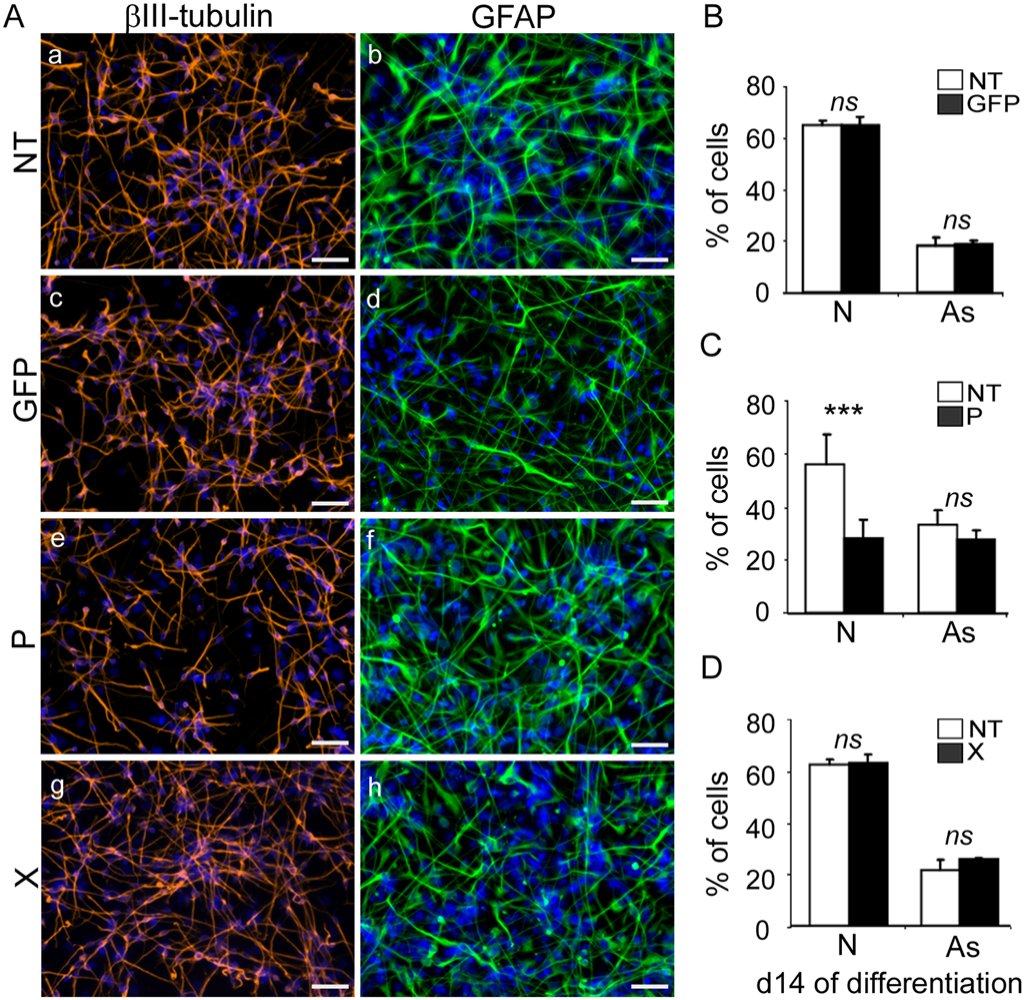
Expression of the *bdv-p* but not *bdv-x* gene in hNPCs impairs neuronal differentiation. Transduced hNPCs expressing *gfp*, *bdv-p* or *bdv-x* genes and their matched NT controls were induced to differentiate for 14 days. (A) Immunostaining with antibodies directed against βIII-Tubulin, a neuronal marker (red), or GFAP, an astrocytic marker (green). Nuclei were stained with DAPI (blue). For panel homogenization, GFAP immunostaining performed in *gfp*-expressing hNPCs was re-colored in green. The percentage of neurons and astrocytes was determined based on enumeration of βIII-Tubulin-, GFAP- and DAPI-positive cells in (B) *gfp*-expressing hNPCs, (C) *bdv-p*-expressing hNPCs and (D) *bdv-x*-expressing hNPCs. Results in B, C and D are representative of 2, 5 and 2 independent experiments, respectively. All experiments were performed in triplicate. Statistical analyses were performed using the Mann-Whitney test. ***, p < 0.001, ns, non-significant (p > 0.5). N, neurons. As, astrocytes. Scale bar, 50 μm.

When growth factors are withdrawn from the medium, hNPCs differentiate into a mixed culture composed mainly of neurons and astrocytes. Very few oligodendrocytes are produced in our conditions of culture. To evaluate the effect of viral *bdv-p* and *bdv-x* gene on neural differentiation, transgene-expressing hNPCs and their NT matched controls that had undergone differentiation for 14 days were fixed and immunostained with antibodies directed against the neuronal marker βIII-Tubulin and the astroglial marker glial fibrillary acidic protein (GFAP). In keeping with our previous findings, differentiated NT hNPCs exhibited a pattern typical of mixed culture, being composed of 55% to 65% of neurons and 20% to 30% of astrocytes (Fig 3, non-transduced). No difference was observed in the percentage of either neurons or astrocytes generated in *gfp*-expressing hNPCs as compared with their matched NT controls (Fig 3A, 3Aa–3Ad and 3B). Thus, lentiviral transduction *per se* did not affect differentiation of hNPCs. We then evaluated the impact of *bdv-p* and *bdv-x* genes. In *bdv-p*-expressing hNPCs, we observed a decrease of up to 50% of neurons upon enumeration of immunostained cells (Fig 3A, 3Aa, 3Ae and 3C, left). A similar result was obtained when cells were immunostained with an antibody directed against a second neuronal marker, the microtubule-associated protein 2 (MAP2), (S2A and S2B Fig), thus confirming the observed diminution in the percentage of neurons. By contrast, the percentage of astrocytes was not modified (Fig 3A, 3Ab, 3Af and 3C, right). In *bdv-x*-expressing hNPCs, neither neurons nor astrocytes were altered, as their pattern and percentage were similar to those observed in their matched NT controls (Fig 3Aa, 3Ag, 3Ab, 3Ah and 3D). Thus, our results demonstrate that expression of the viral *bdv-p* but not *bdv-x* gene alters neuronal differentiation but spares the astrocytic lineage.

### The *bdv-p*-induced decrease in neurons is not due to cellular death

**Fig 4:**
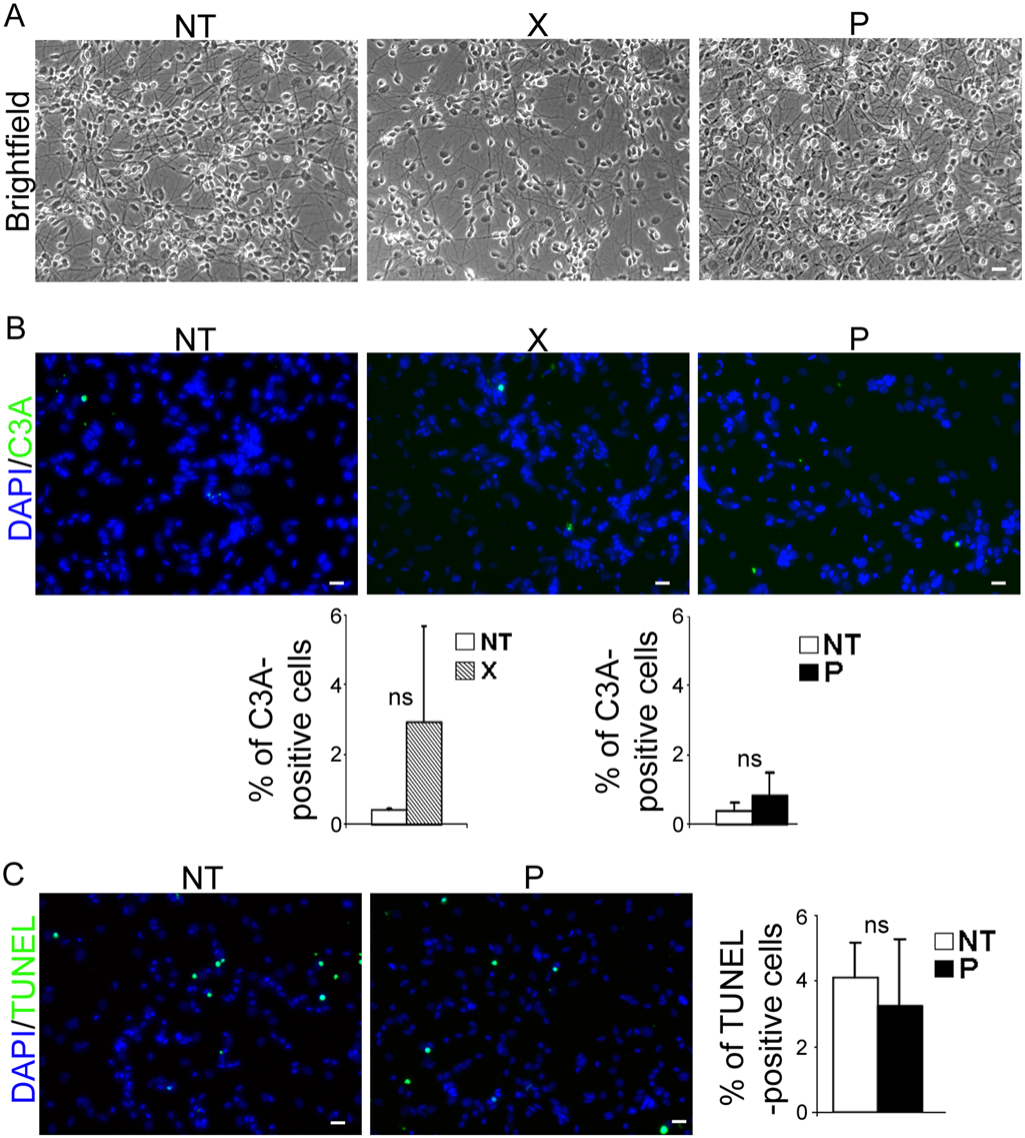
*bdv-p* expression does not induce cellular death in differentiated hNPCs. Transduced hNPCs expressing *bdv-p* or *bdv-x* genes and their matched NT controls were induced to differentiate for 14 days and observed by (A) phase-contrast microscopy. Apoptosis was quantified based on (B) immunostaining with an antibody directed against cleaved-caspase 3 (green) and (C) TUNEL assay (green). Nuclei were stained with DAPI (blue). The data represent the mean of 2 independent experiments performed in triplicate. Statistical analyses were performed using the Mann-Whitney test. *ns*, non-significant (p > 0.5). Scale bar, 20 μm.

The decrease in the number of neurons in *bdv-p*-expressing hNPCs may have been due to increased death of cells committed to neuronal fate, or alternatively, to blockade in neuronal differentiation. To address the first possibility, we sought evidence of a cytopathic effect or apoptosis. Observation by phase-contrast microscopy at 4, 7, 10 and 14 days of differentiation did not reveal any obvious cytopathic effect in either *bdv-p*- or *bdv-x*-expressing hNPCs, as compared with their matched NT controls (Fig 4A, day 14). Apoptosis was sought in cells differentiated for 14 days by immunostaining using an antibody directed against cleaved caspase 3, a well-known apoptotic marker, and by TUNEL assay (Fig 4B and 4C). Observation and enumeration of cleaved-caspase-3- (Fig 4B) and TUNEL- (Fig 4C) positive cells revealed very few apoptotic cells in hNPCs, whether NT or expressing *bdv-x* or *bdv-p*. This showed that P did not compromise the survival of differentiating cells. Thus, P-induced reduction in the number of neurons appears to be due to the decreased capacity of hNPCs to differentiate into neurons rather than to impairment of their survival.

### Expression of the *bdv-p* gene in hNPCs does not impair neuronal specification

**Fig 5:**
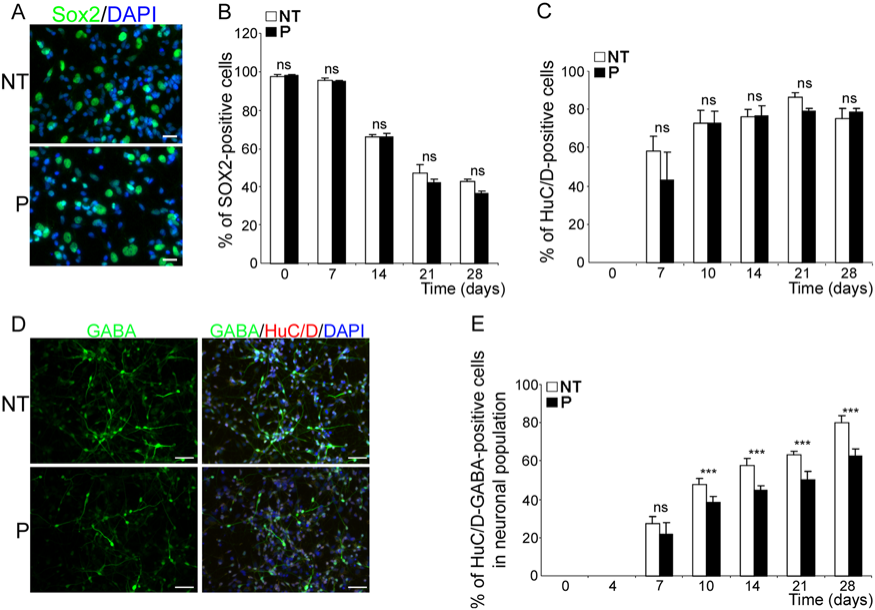
*bdv-p* expression does not alter neuronal specification but induces a reduction in the GABAergic subpopulation. Transduced hNPCs expressing *bdv-p* and their matched NT controls were induced to differentiate for 0, 7, 10, 14, 21 and 28 days and immunostained with antibodies directed against markers of different stages of differentiation. (A) immunostaining of hNPCs differentiated for 28 days with an anti-Sox2 antibody (green). Nuclei were counterstained with DAPI (blue). Scale bar, 20 μm. Time-course analyses showing the percentage of (B) Sox2-positive cells and (C) HuC/D-positive cells. (D) Immunostaining of hNPCs differentiated for 14 days with antibodies against HuC/D and GABA. Nuclei were stained with DAPI (blue). Scale bar, 50 μm. (E) Time-course analysis showing the percentage of huC/D- and GABA-positive cells in the total neuronal population. Results are representative of 2 (B) and 3 (C and E) independent experiments performed in triplicate. Statistical analyses were performed using the Mann-Whitney test. ***, p < 0.005. *nd*, non-determined.

To define the stage at which neuronal differentiation was impaired with greater precision, we performed a time-course study in which the number of Sox2- and HuC/D-positive cells was monitored throughout differentiation. Sox2 is a universal marker of neural progenitors that is known to be down-regulated during differentiation when progenitors become post-mitotic [36]. We reasoned that if a pool of *bdv-p*-expressing hNPCs were blocked at the progenitor stage, Sox2-positive cells would be more numerous than in control cells. We thus labeled *bdv-p*-expressing hNPCs and their NT matched controls with an antibody directed against Sox2 from 0 to 28 days of differentiation. As expected, at the progenitor stage (day 0), 100% of NT hNPCs were Sox-2 positive and their number continuously decreased during differentiation (Fig 5A and 5B). They represented approximately 60% of the population at day 14 and 40% at day 28. It was somewhat surprising to observe that as many as 60% of hNPCs still expressed Sox2 after 14 days of differentiation, since at that time approximately 90% of the cells had already differentiated into either βIII-Tubulin- or GFAP-positive cells. This indicated that loss of Sox2 expression is gradual during the differentiation process. Most notably, no difference was observed in the percentage of Sox2-positive cells between *bdv-p*- and NT- cells at any time point studied, indicating that P does not prevent the cells from exiting the progenitor stage. Next, to address whether P blocks cell entry into the neuronal pathway, *bdv-p*-expressing hNPCs and their NT matched controls were labeled with an antibody directed against HuC/D, a nuclear neuronal marker that is expressed as soon as the neuroblasts exit the proliferation cell cycle [37]. In NT cells, approximately 60% of cells were HuC/D-positive at day 7 and their number rose up to day 10, at which time it remained constant up until day 28 (Fig 5C). This showed that by day 10, commitment to the neuronal lineage has been completed. The estimate of the neuronal population based on HuC/D immunostaining at 14 days of differentiation in NT cells was somewhat higher (approximately 80%) than that previously determined on the basis of βIII-Tubulin immunostaining (approximately 60%, Fig 3). This is possibly due to variability between experiments and/or to differences in the manner in which cells were enumerated (automatically for HuC/D and manually for βIII-Tubulin). To address this issue, HuC/D- and βIII-Tubulin-positive cells were both manually enumerated in one single experiment, at day 14 of differentiation. Similar values were obtained (approximately 70%), indicating that, at that time, all NT cells that had entered a neuronal pathway had acquired the βIII-Tubulin marker. Most notably, at every time point studied, no significant difference in the percentage of HuC/D-positive cells was observed between *bdv-p*-expressing cells and their NT controls (Fig 5C), indicating that P does not impair neuronal commitment. Thus, altogether, our results suggest that P impairs the acquisition of a mature neuronal phenotype.

### Expression of the *bdv-p* gene in hNPCs reduces GABAergic neurogenesis

We then sought to define the neuronal subpopulations that were altered in *bdv-p*-expressing cells. Depending on the age of the fetus, the area of the brain from which cells were collected and the composition of the culture medium, GABAergic and glutamatergic neurons may be produced. To determine which subtypes were present in our cultures, we used the antibody directed against HuC/D for recognition of all neuronal subtypes and antibodies specifically directed against either GABAergic or glutamatergic neurons. We also used an antibody specific for dopaminergic neurons. At day 28 of differentiation, approximately 80% of the neurons were GABAergic, 0.3% were dopaminergic and none of them were glutamatergic (S3 Fig). It is unlikely that the lack of glutamatergic neurons in our cultures is attributable to defective antibodies because two of the three antibodies we tested are routinely used for staining glutamatergic neurons generated from human embryonic stem (hES) cells [38]. Thus, our cultures were predominantly composed of GABAergic neurons. In a time-course study, we verified whether acquisition of the GABAergic phenotype was impaired in *bdv-p*-expressing cells and at which time (Fig 5D and 5E). At the earliest time point examined (day 4), the GABAergic phenotype had not yet been acquired in either NT or *bdv-p*-expressing cells. In NT cells, it was first observed at day 7, at which time GABAergic neurons constituted approximately 30% of the total neuronal population. Their percentage continuously rose to reach approximately 80% at day 28 (Fig 5D and 5E). In *bdv-p*-expressing cells, while the number of GABAergic neurons also continuously rose from day 7 to day 28, their proportion was reduced throughout differentiation, as compared with that of NT cells (Fig 5E). Thus, *bdv-p* expression in differentiating hNPCs diminishes GABAergic neurogenesis. On the contrary, from day 7 to day 28 of differentiation, the percentage of GABAergic neurons was similar in *bdv-x*-expressing cells and their matched NT controls (S4A and S4B Fig), confirming that BDV-X does not impair human neurogenesis and further demonstrating the specificity of *bdv-p*-induced alteration.

### The S26/28 phosphorylation site in P is not essential for reduction in GABAergic neurogenesis

**Fig 6:**
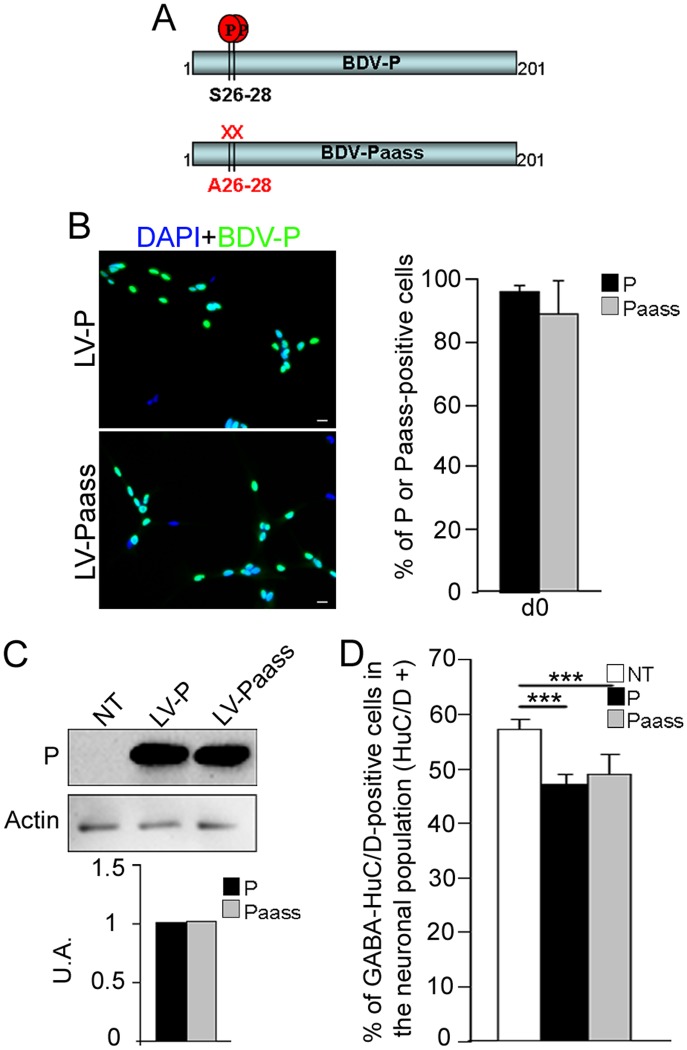
The S26/28 phosphorylation site is not necessary for *bdv-p*-induced reduction in GABAergic neurogenesis. (A) Schematic representation of P and Paass. Paass is mutated in S26/28 residues and thus lacks the corresponding phosphorylation site. (B) hNPCs were transduced with lentiviral vectors bearing the *bdv-p* or *bdv-p*_*aass*_ gene and immunostained at the undifferentiated stage (day 0) with an antibody directed against the P protein (green). Nuclei were stained with DAPI (blue). Transduction efficiency was evaluated by enumeration of P-positive cells (right panel). (C) Western blot showing the level of P in *bdv-p* and *bdv-p*_*aass*_-expressing hNPCs at 14 days of differentiation. P is normalized to actin. (D) Transgenic hNPCs and their NT matched controls were induced to differentiate for 14 days and GABAergic neurons were quantified. The results are representative of 2 independent experiments performed in triplicate. Statistical analyses were performed using the Mann-Whitney test. ***, p = 0.002. Scale bar, 20 μm.

The serine (S) residues at position 26 and 28 (S26 and S28) are known to be phosphorylated by PKC and to form the major site of phosphorylation in the phosphoprotein P [39]. Recently, two atypical PKC, zeta and iota, have been shown to facilitate neuronal differentiation [40]. This prompted us to investigate whether S26 and S28 might be involved in *bdv-p*-induced alteration of GABAergic neurogenesis through alteration of PKC signaling. To address this question, we used a modified P protein in which the S residues were replaced by alanine (A) residues (Fig 6A). In the modified protein, called Paass, P phosphorylation by PKC was shown to be completely abolished [34]. Using lentiviral vectors as previously described, we established new transgenic hNPCs expressing either the *bdv-p* or *bdv-p*_*aass*_ gene. A similar percentage of cells was transduced in the two transgenic lines, as 96 +/- 2% and 87 +/- 5% of cells expressing the *bdv-p* or *bdv-p*_*aass*_ gene, respectively, were P-positive (Fig 6B). Both cellular localization and level of expression of P were also comparable in the two cell lines, as shown by immunofluorescence (Fig 6B) and Western blotting (Fig 6C). The *bdv-p*- and *bdv-p*_*aass*_-expressing hNPCs, together with their matched NT controls, were induced to differentiate for 14 days and the percentage of GABAergic neurons generated was determined. The phosphorylation status of S26/28 residues clearly did not modify P-induced inhibition of GABAergic neurogenesis, as a similar percentage of GABAergic neurons was generated in *bdv-p-* and *bdv-p*_*aass*_-expressing hNPCs. In both cases, the percentage was significantly lower than in the NT matched controls (Fig 6D). These results establish that the phosphorylation of the serine residues S26/28 is not essential for P-induced impairment of GABAergic neurogenesis.

### Expression of the *bdv-p* gene in hNPCs alters the molecular program that controls neurogenesis

**Fig 7:**
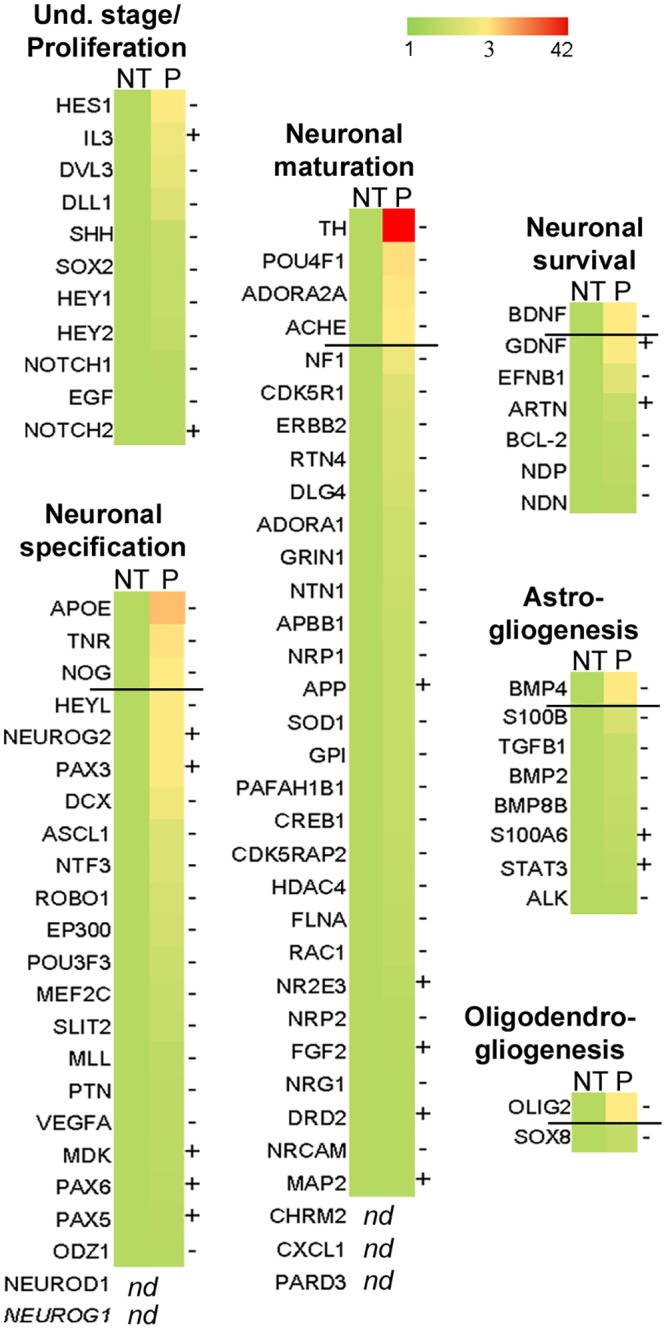
*bdv-p* alters the molecular program involved in neurogenesis. *bdv-p*-expressing-hNPCs and their matched NT controls were induced to differentiate for 4 days and RNA was analyzed using an RT-PCR array. The 84 human genes analyzed are shown. A color code shows the differential expression of the genes, from green (non-regulated) to red (the most regulated). Genes were grouped depending on their known function in neurogenesis. Up to the dark line are the genes significantly modified after application of an arbitrary cut-off of 3. -, genes down-regulated. +, genes up-regulated.

Neuronal differentiation is tightly regulated by an intrinsic genetic program. To determine whether this program was impaired in *bdv-p*-expressing hNPCs, we analyzed the differential expression of 84 human genes involved in neural differentiation, using a PCR array approach. Under the assumption that transcriptional alterations should precede cellular alterations, transcripts from *bdv-p*-expressing hNPCs were pooled from biological triplicates and compared with their matched NT controls at an early time point of differentiation (day 4). All genes studied are shown in Fig 7 and genes significantly modulated, after application of an arbitrary cut-off of 3-fold, are listed in Table 1. In confirmation of our finding that *bdv-p* expression had no effect at the undifferentiated stage, the genes involved in proliferation and maintenance of hNPCs were not significantly regulated in *bdv-p*-expressing cells. In contrast, 10 genes known to be involved in either neuronal specification (*ApoE*, *Tnr*, *noggin*) or neuronal maturation and survival (*TH*, *Pou4f1*, *Adora2A*, *Bdnf*, *AchE*) or glial differentiation (*Bmp4*, *Olig2*) were down-regulated. Among these, tyrosine hydroxylase (*TH*) and apolipoprotein E (*ApoE*) were the most dramatically regulated, with a 42- and 10-fold decrease, respectively.

**Table 1:** Genes modulated in *bdv-p*-expressing hNPCs.

Gene symbol	Encoded protein	Fold change (P/NT)	Function
TH	Tyrosine Hydroxylase	- 42,5	Dopaminergic neuron marker
ApoE	Apolipoprotein E	- 10,1	Promotes neurogenesis
Pou4f1	POU-domain class 4 transcription factor 1	- 5,3	Promotes neurite outgrowth and neuroprotection
Tnr	Tenascin R	- 4,5	Promotes neuronal differentiation and maturation
Adora2A	Adenosine A2A Receptor	- 3,9	Promotes neuronal maturation
Bmp4	Bone Morphogenetic Protein 4	- 3,5	Promotes astrocyte differentiation
Bdnf	Brain-Derived Neurotrophic Factor	- 3,2	Promotes neuronal maturation and neuroprotection
AchE	Acetylcholinesterase	- 3,2	Promotes neurite outgrowth
Nog	Noggin	- 3,1	Promotes neurogenesis
Olig2	Oligodendrocyte Lineage Transcription Factor 2	- 3,1	Promotes oligodendrocyte differentiation

PCR array analysis of genes differentially expressed in *bdv-p*-expressing hNPCs differentiated for 4 days compared with their non-transduced (NT) matched controls.

**Fig 8:**
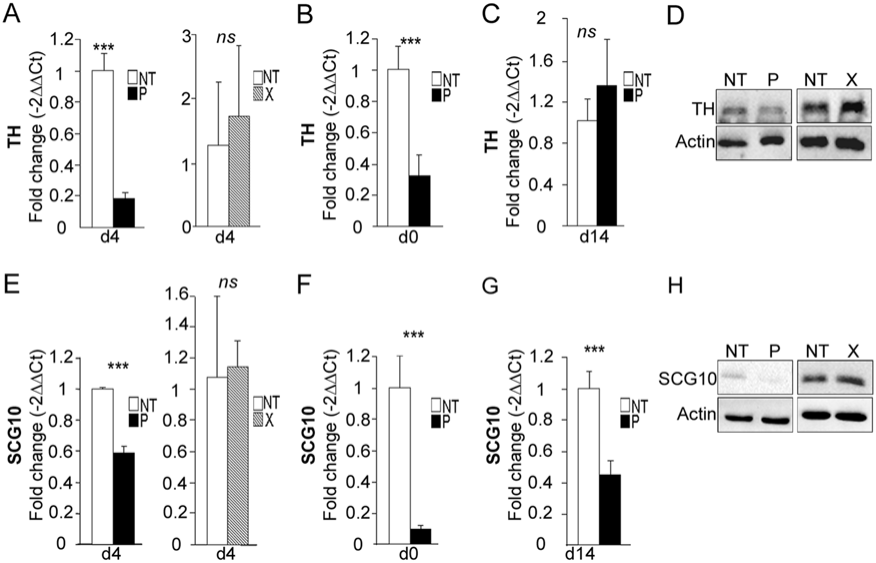
*bdv-p* alters the expression of *TH* and *Scg10/Stathmin2*. *bdv-p*- and *bdv-x*-expressing- hNPCs and their matched NT controls were induced to differentiate for 0, 4 or 14 days before RNA and protein analyses. *TH* expression was measured by RT-qPCR at (A) day 4, (B) day 0 and (C) day 14. (D) Western blot analysis showing TH level. *Scg10/Stathmin2* expression was measured by RT-qPCR at (E) day 4, (F) day 0 and (G) day 14. (H) Western blot analysis showing SCG10/Stathmin2 level. TH and SCG10/Stathmin2 were normalized to actin. The results are representative of 2 independent experiments performed in triplicate. Statistical analyses were performed using the Mann-Whitney test. ***, p < 0.001.

TH is a well-known marker of dopaminergic neurons, although it is also expressed to a lesser extent in neural progenitor cells, in which its function remains uncertain. In order to validate our PCR array data, primers were specifically designed to re-analyze its expression by RT-qPCR on individual samples. Down-regulation of *TH* mRNA in *bdv-p*-expressing hNPCs differentiated for 4 days was confirmed, although with a more modest 5-fold decrease (Fig 8A, left). In contrast, there was no modification in the *TH* mRNA level in *bdv-x*-expressing hNPCs (Fig 8A, right), demonstrating that down-regulation was specifically induced by the P protein. Decrease in *TH* mRNA occurred as early as day 0, in undifferentiated *bdv-p*-expressing hNPCs (Fig 8B), but this difference was no longer observed at 14 days of differentiation (Fig 8C). At day 4, P-induced down-regulation was confirmed at the protein level, as shown by Western blotting (Fig 8D). In contrast and as expected, X did not alter the level of TH protein (Fig 8D, right).

TH, BDNF and SCG10/Stathmin2 are three pro-neuronal factors that are known to be regulated by REST/MeCP2 signaling, a major pathway in neurogenesis [41,42]. As we observed a down-regulation in *TH* and *Bdnf* in the PCR array, we wondered whether *Scg10*/S*tathmin2*, which had previously been shown to be altered in BDV-infected rat hippocampal neurons [43], was also down-regulated in *bdv-p*-expressing hNPCs. This was indeed the case, as a 1.8-fold decrease was observed (Fig 8E, left). Again, no alteration was shown in *bdv-x*-expressing hNPCs (Fig 8E, right). Like *TH*, *Scg10*/S*tathmin2* down-regulation was observed before the initiation of differentiation, at day 0 (Fig 8F), but in contrast to TH it was durable and still evident at day 14 (Fig 8G). This was confirmed at the protein level (Fig 8H). To determine whether down-regulation of genes regulated by REST and MeCP2 might result from a modification of their expression, we evaluated *REST* and *MeCP2* levels in *bdv-p*-expressing hNPCs. RT-qPCR and Western blotting analyses, however, did not reveal any alteration at mRNA or protein levels at any time point studied (S5A–S5D Fig for REST and S5E–S5H Fig for MeCP2).

**Fig 9:**
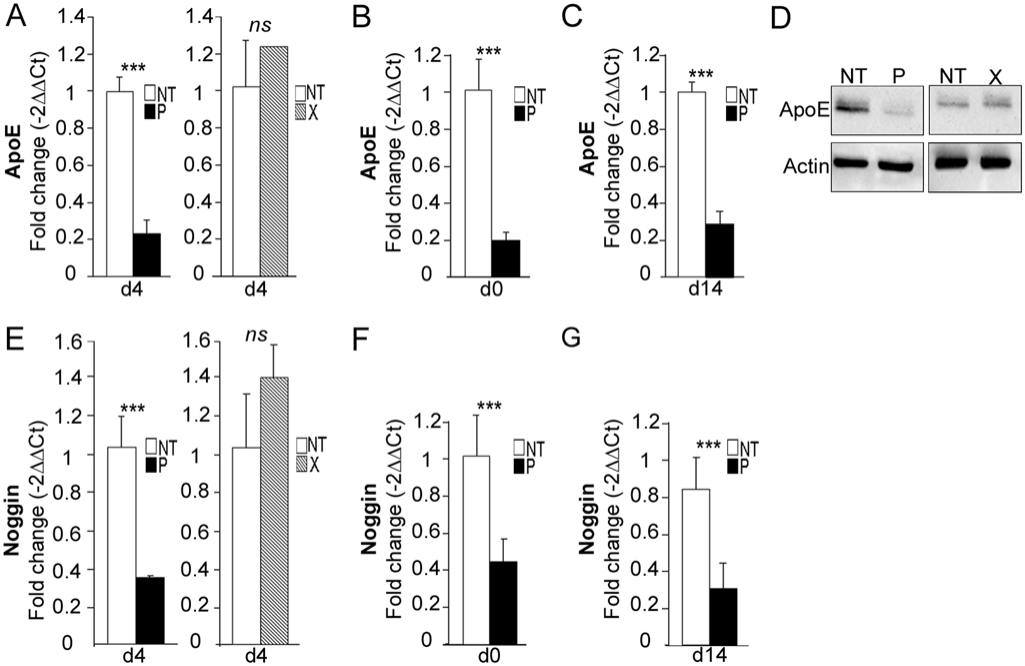
*bdv-p* alters the expression of *ApoE* and *Noggin*. *bdv-p*- and *bdv-x*-expressing-hNPCs and their matched NT controls were induced to differentiate for 0, 4 or 14 days before RNA and protein analyses. *ApoE* expression was measured by RT-qPCR at (A) day 4, (B) day 0 and (C) day 14. (D) Western blot analysis showing ApoE level. It was normalized to actin. *Noggin* expression was measured by RT-qPCR at (E) day 4, (F) day 0 and (G) day 14. The results are representative of 2 independent experiments performed in triplicate. Statistical analyses were performed using the Mann-Whitney test. ***, p < 0.001.

*ApoE* was the second most down-regulated gene (10-fold decrease) in *bdv-p*-expressing hNPCs. In a study by Li *et al*. [44], it was shown that invalidation of the *ApoE* gene was linked to decrease in noggin and to alteration in neurogenesis. As *Noggin* was also shown to be decreased in the PCR array (by a 3-fold factor, Fig 7 and Table 1), we sought to confirm the down-regulation of these two genes by RT-qPCR. Down-regulation of *ApoE* was clearly confirmed at day 4 of differentiation, as a 4.4-fold decrease was observed (Fig 9A, left). In contrast, as already shown for *TH* and *Scg10/Stathmin2*, there was no alteration in *bdv-x*-expressing hNPCs (Fig 9A, right). Also, similar to observations made for *Scg10/Stathmin2*, down-regulation of the *ApoE* gene occurred before the initiation of differentiation, at day 0 (Fig 9B), and was still observable at day 14 (Fig 9C). It was also further confirmed at the protein level (Fig 9D), with again, no alteration due to the X protein. Similar results were obtained for *noggin* as a 2.9-fold decrease occurred at day 4 and was observable at day 0 and day 14 of differentiation (Fig 9E, 9F and 9G).

Taken together, our results demonstrate that P impairs the developmental program involved in hNPC differentiation and strongly suggest certain genes to be responsible for *bdv-p*-induced inhibition of neurogenesis.

## Discussion

BDV alters the behavior of infected hosts by mechanisms that remain largely unknown. Recently, we have uncovered a new mechanism by which it may do so, as we demonstrated its capacity to impair human neurogenesis [29]. Here, we sought to identify the viral proteins and molecular pathways that are responsible for BDV impairment of neurogenesis. We demonstrate that the phosphoprotein P, but not the X protein, reduces human neurogenesis and affects GABAergic neurons. In addition, we reveal an alteration in expression of pro-neuronal factors controlling neurogenesis that precedes and accompanies cellular dysfunction.

Using specific markers, we have defined the stage at which neuronal differentiation is impaired by P. We show that *bdv-p*-expressing hNPCs exit the cell cycle and enter the neuronal pathway, suggesting that P expression impairs the acquisition of a mature neuronal phenotype, including expression of βIII-Tubulin, MAP2 and GABA markers. This reduction in neurogenesis was not accompanied by cellular death, in contrast with our previous findings, which showed extensive cell death in BDV-infected differentiating hNPCs [29]. This difference may be related to the necessity for viral replication or for a combination of viral proteins to induce death. One may speculate, for example, that interactions between P and X would lead to the localization of P in not only the nucleus, as observed in our study, but also in the cytoplasm [33]. This would most likely induce interference with signaling pathways other than those described here, possibly resulting in cell death. Thus, although the mechanisms underlying the cytocidal effect of BDV during neuronal differentiation remain to be fully elucidated, we clearly show that a single protein, the P, is sufficient to reduce neurogenesis.

Gamma-aminobutyric acid (GABA) is the chief inhibitory neurotransmitter that regulates neuronal excitability in the mammalian brain. It is important to note that BDV has been reported to alter this system through different mechanisms. *In vivo*, BDV infection of newborn rats induces a strong reduction in the volume of cortical areas, which has been associated with depletion of GABAergic neurons [45]. *In vitro*, studies have shown that P binds to the GABA receptor-associated protein (GABARAP), a molecule that links the GABA receptor (GABAR) to microtubules, which may lead to alteration in GABAergic synaptic transmission through limitation of GABAR mobility [27]. Finally, our finding that P impairs GABAergic neurogenesis has revealed a new mechanism by which BDV may alter GABAergic neurotransmission in the brain. This is particularly intriguing because abnormalities in the generation of GABAergic neurons are viewed as characteristic of several neuropsychiatric disorders [30,46]. Whether P induces a definitive loss in GABAergic neurons or simply a delay in neuronal maturation could not be determined in our study, as by 28 days of differentiation, the latest time point studied, the neurons had not all acquired the GABAergic phenotype. In both cases, however, brain damage would be expected to occur, as brain development is dependent not only on the acquisition of the proper number of neurons, but also on their acquisition at the proper time. Whether this neuronal subpopulation is affected in the context of BDV infection has not yet been specifically addressed, but appears highly probable, as we have shown that most of the neurons derived from hNPCs are predestined towards a GABAergic fate.

The fate of NPCs is tightly controlled by an intrinsic genetic program that is still incompletely understood, especially in human NPCs. Here, we show that 11 genes known to be involved in neural differentiation are down-regulated in *bdv-p*-expressing hNPCs. Among these, *TH*, *Bdnf* and *Scg10/Stathmin2* are known to be pro-neuronal factors. Their regulation by BDV has been previously observed in rat hippocampal and cortical neurons [43] or neuronal-like PC12 cells [23]. Here, we further show that P is responsible for their regulation in hNPCs. Notably, these three pro-neuronal factors are under the control of the REST/MeCP2 signaling, a pathway known to be critically important for neurogenesis [41, 47–50]. This observation, along with the fact that BDV had been previously shown to regulate MeCP2 expression in rat cortical neurons [43], prompted us to analyze this pathway. P, however, regulated neither *Rest* nor *Mecp2* expression in hNPCs. The difference between our study and that of Suberbielle *et al*. [43] may be explained either by the implication of viral proteins other than P in *Mecp2* regulation or by differences that can be attributed to cell type or species. Nevertheless, it is striking that expression of several pro-neuronal factors that are directly under the control of REST/MeCP2 is altered. It is tempting to suggest that P may induce other modifications in MeCP2 and/or REST that have an impact on their expression. Post-translational modifications in MeCP2 [47,51,52], modification in REST activity through alteration of either the nuclear complex that is necessary for its repressive activity or cellular localization, or differential regulation of its isoforms [53] are all possibilities that represent exciting challenges for the future.

Several studies have concurred that ApoE is involved in adult neurogenesis in mice. In particular, its expression was evidenced *in vivo* in NPCs found in two neurogenic areas, the sub-granular zone (SGZ) of the dendate gyrus (DG) and the sub-ventricular zone (SVZ) [54] and was shown to be up-regulated *in vitro* in murine NPCs induced to differentiate with retinoic acid [55]. *In vitro* analyses using NPCs isolated from adult *ApoE*-/- mice further demonstrated that invalidation of the *ApoE* gene induced a reduction in the number of neurons that were generated during adult neurogenesis [44]. This was accompanied by an 80% decrease in *Noggin* expression, suggesting that Noggin, a factor that is known to facilitate neurogenesis [56], plays a role in this mechanism. These observations, together with our findings showing a decrease in both *ApoE* and *Noggin* expression in *bdv-p*-expressing hNPCs, strongly suggest that the ApoE-Noggin pathway is a mediator of P-induced impairment of human neurogenesis. The underlying mechanisms, however, appear to differ between the study by Li *et al*. [44] and our own. Whereas the decrease in neurogenesis was due to altered neuronal specification and was accompanied by an increase in astrogliogenesis in the Li *et al*. study [44], neuronal specification and astrocyte number were not altered in ours. This may reveal differences between adult and fetal neurogenesis or between rodent and human neurogenesis. Whether the ApoE-Noggin pathway plays a role in the generation of different neuronal subtypes may be questioned. This is suggested by both studies as one addresses glutamatergic neurogenesis while the other focuses on the generation of GABAergic neurons. However, differences in timing and species in the two studies preclude direct comparison and future studies are required to address this issue in human neurogenesis. Another candidate that may potentially play a role in P-induced inhibition of GABAergic neurogenesis is the *tenascin R* (*tnr*) gene. This extracellular matrix glycoprotein has very recently been shown to regulate the genesis of GABAergic neurons [57]. Our observation, by PCR array, that its expression is decreased in *bdv-p*-expressing hNPCs, suggests its involvement in P-induced reduction of neurogenesis. Xu *et al*. [57], however, showed that the deletion of the *tnr* gene was associated with an increase in GABAergic neurons in mice, which is in apparent contradiction to our observation. Further studies thus will be required to evaluate the functional importance of each of these molecules in P-induced reduction in human neurogenesis.

How P is linked to downstream signaling is not yet understood. Several studies have shown that it interferes with cellular signaling by acting as a decoy substrate for cellular kinases. This was shown to have dramatic consequences for the immune response [58] and neuronal transmission [25]. In the latter case, it was established that the damage to synaptic transmission in BDV-infected rat hippocampal neurons was due to P-mediated interference with PKC signaling [59]. Here, we used a modified P protein in which the PKC phosphorylation site had been abrogated and showed that it was not necessary for P-induced impairment of GABAergic neurogenesis. We therefore suggest that the viral P protein interferes with signaling in the brain in another manner, independent of PKC signaling. Identification of the missing factor that interacts with P and links it to downstream signaling would cast light on the mechanisms by which the viral phosphoprotein P interferes with brain function.

The development of hNPC cultures has allowed the direct impact of viruses on human neurogenesis to be addressed, a process that is fundamental for both brain development and normal brain function in the adult. Several neurotropic viruses, including BDV, are now known to affect human neurogenesis [29, 60]. In this study, we provide the first evidence that a viral protein, the phosphoprotein P, impairs GABAergic neurogenesis, a pathological process that is characteristic of several psychiatric disorders. This result strengthens the view that persistent viral infection of the CNS may play a role in human mental disorders, as has been suggested by others [8]. Although future studies will have to be conducted to describe in full the mechanisms by which this occurs, we provide the first molecular clues as to how a viral protein impairs the genetic program that leads to neuronal differentiation. Our study thus improves our understanding of the mechanisms by which BDV interferes with brain function and identifies an original molecular tool, the phosphoprotein P, that can be used to characterize the process by which human GABAergic neurons are generated. Our findings may contribute to a better understanding of psychiatric disorders and to development of improved therapies in the future.

## Materials and Methods

### Ethics statement

Human fetuses were obtained after legal abortion with written informed consent of the patient. The procedure for the procurement and use of human fetal CNS tissue was approved and monitored by the “Comité Consultatif de Protection des Personnes dans la Recherche Biomédicale” of Henri Mondor Hospital, France.

### hNPC culture

hNPCs were prepared and cultured as previously described in [29].

### Lentiviral vector production and transduction of hNPCs

The genes encoding the viral P and X proteins or green fluorescent protein (GFP) were amplified by PCR and cloned into the pTrip lentiviral vector backbone downstream of the constitutive cytomegalovirus (CMV) enhancer/chicken ß-actin (CAG) promoter (a kind gift of Dr. P. Charneau, Pasteur Institute, Paris, France), using *BamH*I and *Xho*I restriction sites. To produce the lentiviral vectors, 10 T150 flasks plated with 1.2 x10^7^ HEK-293T cells each were cotransfected with the two packaging plasmids, psPAX2 and pMD2.G (Addgene, France), and the pTrip plasmid expressing one of the genes of interest (14.6, 7.9 and 22.5 μg of each endotoxin-free prepared plasmids were used, respectively) mixed with 100 μl of GeneJuice (Merck, France). Culture medium was removed the next day and replaced by warm OptiMEM medium (Gibco, France). Supernatants were collected 48 h and 72 h post-transfection, cleared by low-speed centrifugation and filtered using a 0.45 μm filter. Lentiviral particles were then purified by ultracentrifugation through a 20% sucrose cushion (25,000 rpm, 2 h, 4°C; SW32Ti rotor, Beckman Coulter). Pellets were resuspended in ice-cold PBS under gentle agitation overnight at 4°C, aliquoted and stored at -80°C. Titers of the lentiviral vectors were determined by counting foci 72 h after transduction of HEK-293T cells with serial dilutions of vectors. In all our experiments, titers varied from 8x10^8^ to 3x10^9^ transduction units (TU) ml^-1^ and lentivectors were used at a multiplicity of transduction (transduction units of vector for each cell) of 1 to 10.

### Neuronal and glial differentiation

Three to five passages after transduction, transduced hNPCs and their matched NT controls were plated in 24-well plates or 6 cm-dishes at a density of 53000 cell/cm^2^ and induced to differentiate upon withdrawal of EGF and bFGF (Abcys, Eurobio, France), as previously described [29]. Of note, as the cell density at plating determines the percentage of each cellular types generated upon differentiation [61], special care was taken to ensure homogeneous cell numbers. In particular, the number of plated transduced and NT hNPCs was systematically verified by counting cells at day 0 of differentiation (DAPI staining, 1 μg/ml, Life technologies, France). Experiments in which the number of cells varied for different conditions were discarded.

### Proliferation test

Two tests were used to quantify hNPC proliferation in the presence of growth factors, EGF and bFGF. The Wst1 Cell Proliferation Assay (Roche, France) was used as described previously [29], except that transduced hNPCs and their matching NT controls were plated on 48-well plates pre-coated with matrigel (Corning, France), at a density of 5000 cells/well. For the Cell Proliferation ELISA, BrdU kit (Roche, France), transduced hNPCs and their matched NT controls were plated at a density of 15000 cells/well in 96-well plates (Falcon, Corning, France) and maintained in proliferation for 4 days before addition of BrdU for 5 h at 37°C. Experiments were then processed according to the manufacturer’s instructions. To analyze hNPC proliferation in the absence of growth factors (early phase of differentiation), transduced hNPCs and their matched NT controls were plated in 48-well plates at a density of 40000 cells/well and stained with DAPI at day 0 and day 4 of differentiation. Images were acquired from 4 fields per well and an average of 130 cells per well were counted.

### Antibodies

Primary antibodies were as follows: anti-βIII-Tubulin (mouse monoclonal, Sigma, France), anti-βIII-Tubulin (rabbit polyclonal, Abcam, France), anti-actin (mouse monoclonal, Sigma, France), anti-ApoE (goat polyclonal, Millipore, France), anti-Cleaved caspase-3 (rabbit polyclonal, Cell Signaling, France), anti-GABA (rabbit polyclonal, Sigma, France), anti-GFAP (rabbit polyclonal, DAKO, France), anti-vGLUT1 (rabbit polyclonal, Abcam, France) anti-glutamate transporter neuronal (EAAC1) (goat polyclonal, Millipore, France), anti-vGLUT1 and anti-vGLUT2 (rabbit polyclonal, Synaptic Systems, Germany), anti-HuC/D (mouse monoclonal, Molecular Probes, Life Technologies, France), anti-MAP2 (mouse monoclonal, Sigma, France), anti-MeCP2 (rabbit, a generous gift from Dr E. Joly, Toulouse, France), anti-REST (rabbit polyclonal, Millipore, France), anti-SCG10 (rabbit polyclonal, a generous gift from Dr A. Sobel, Paris, France), anti-TH (rabbit polyclonal, Abcam, France), anti-SOX2 (rabbit polyclonal, Millipore, France) and anti-BDV-P and anti-BDV-X (rabbit polyclonal antibody).

### Immunofluorescence analyses

Transgene-expressing hNPCs and their matched NT controls were plated at a density of 53,000 cell/cm^2^ on 24-well plates optimized for automated image acquisition (Ibidi, France). Undifferentiated and differentiated hNPCs were fixed 20 min in 4% paraformaldehyde (Electron Microscopy Sciences, France) and standard immunofluorescence was performed as described previously, for βIII-Tubulin, MAP-2, GFAP, cleaved-caspase 3 and BDV-P antibodies [29]. For all other antibodies, cells were blocked for 1h in PBS-3%BSA and primary antibodies were incubated in PBS-0.1%Triton-X-100-1%BSA overnight at +4°C. Secondary antibodies were anti-mouse IgG coupled with Alexa Fluor-546, or anti-rabbit IgG coupled with Alexa Fluor 488 or 647 (Molecular Probes, Invitrogen, France). Nuclei were stained with DAPI. Images of cells immunostained with βIII-Tubulin, GFAP or MAP2 antibodies were acquired with the AxioObserver Z1 inverted microscope (Zeiss) using either Axiovision or ZEN software (Zeiss). In every experiment, six wells per condition were analyzed and an average of 1,000 cells per well were manually enumerated. Images of cells immunostained with HuC/D, GABA and Sox2 antibodies were acquired using the Cellomics ArrayScan automated microscope (Thermofisher Scientific, France) and cell enumeration was automatically performed. In every experiment, six wells per condition were analyzed and an average of 3,000 cells per well were enumerated.

### PCR array

Total RNA was extracted using the RNeasy mini kit (Qiagen, France) according to the manufacturer’s instructions. Five hundred nanograms of RNA were reverse-transcribed with the RT² First Strand Kit (SA Biosciences, Qiagen, France) and cDNA was subjected to a PCR array specific for human neurogenesis (RT^2^ Profiler PCR array—Human Neurogenesis PAHS-404ZA, SA Biosciences, Qiagen, France). The manufacturer's instructions were strictly followed for reverse transcription and PCR array. Genes were analyzed from RNA pooled from biological triplicates for each condition. Data were normalized using 5 house-keeping genes and analyzed using the -2ΔΔCt method for relative quantification.

### Reverse transcription and quantitative real—time PCR

Total RNA was extracted using Nucleospin RNA/protein (Macherey Nagel, France) according to the manufacturer’s instructions. Reverse transcription and quantitative PCR were performed as described previously [29]. Primers used are listed in S1 Table. PCR efficiencies ranged between 93% and 100%, depending on primer pairs. For relative quantification, the -2ΔΔCt method was used. The reference gene was *Gapdh* and each gene was analyzed from two independent experiments performed in triplicate.

### Western blot

Proteins and RNA were extracted from the same biological samples using the Nucleospin RNA/protein kit (Macherey Nagel, France). Proteins were quantified using the Protein Quantification Assay kit (Macherey Nagel, France), according to the manufacturer’s instructions. Western blot analyses were performed as described previously [29] except that blots were blocked for 1h at RT in PBS-0.1%Tween-20-5% dry milk.

### Accession numbers

The GenBank (www.ncbi.nlm.nih.gov/Genbank) accession numbers for genes mentioned in the article are ApoE (NM_000041), Gapdh (NM_002046), MeCP2 (NM_004992), nestin (NM_006617), Nog/Noggin (NM_005450), Rest (NM_005612), Scg10 (NM_007029), Sox2 (NM_003106), Th (NM_000360). The accession numbers for proteins cited in the text are ApoE (AAH03557), BDV Phosphoprotein (BDV-P, P0C798), BDV X protein (Q912Z9), GFAP (AAB22581), HuC (NP_001411), HuD (NP_001138246), MAP2 (AAB 48098), MeCP2 (P51608), REST (NP_005603), SCG10 (AAB36428), Sox2 (NP_003181), and TH (P07101).

### Statistical analysis

Data represent the means ± standard deviation (SD). Statistical analyses were performed using the Mann-Whitney test. ***, p < 0.001 or p < 0.005 depending on experiments; *ns*, non-significant (p > 0.05).

## Supporting Information

S1 Fig Expression of *gfp*, *bdv-p* and *bdv-x* does not alter the morphology and the survival of hNPCs. Representative phase-contrast photomicrographs of NT, *gfp-*, *bdv-p*- and *bdv-x*-expressing hNPCs at undifferentiated stage. Scale bar, 50 μm.
(TIF)

S2 Fig *bdv-p* impairs neuronal differentiation. *bdv-p*-expressing hNPCs and their matched NT controls were induced to differentiate for 14 days and (A) immunostained with an antibody directed against MAP2, a neuronal marker (red). Nuclei were stained with DAPI (blue). (B) The percentage of neurons was determined based on enumeration of MAP2-positive cells. Results are representative of 2 independent experiments performed in triplicate. Statistical analysis was performed using the Mann-Whitney test. ***, p < 0.001, ns, non-significant (p > 0.5). Scale bar, 50 μm.
(TIF)

S3 Fig Neuronal subtypes in differentiated hNPCs. Non-transduced hNPCs were induced to differentiate for 28 days and (A) immunostained with antibodies directed against HuC/D (total neurons, red), GABA (GABAergic neurons, green), TH (dopaminergic neurons, green) and v-glut1/2 (glutamatergic neurons, green). Nuclei were counterstained with DAPI (blue). (B) Percentage of neuronal subtypes. Results are representative of 2 independent experiments performed in triplicate. Scale bar, 50 μm.
(TIF)

S4 Fig *bdv-x* expression does not reduce the GABAergic subpopulation. *bdv-x*-expressing hNPCs and their matched NT controls were induced to differentiate for 7, 14, 21 and 28 days and immunostained with antibodies directed against HuC/D (total neurons, red) and GABA (GABAergic neurons, green). (A) hNPCs differentiated for 14 days. Nuclei were counterstained with DAPI (blue). Scale bar, 50 μm. (B) Time-course analysis showing the percentage of GABAergic neurons in the total neuronal population. Results are representative of 2 independent experiments performed in triplicate. Statistical analyses were performed using the Mann-Whitney test. ***, p < 0.005, *nd*, non-determined.
(TIF)

S5 Fig *bdv-p* does not alter the expression of *Rest* and *Mecp2*. *bdv-p*- and *bdv-x*-expressing hNPCs and their matched NT controls were induced to differentiate for 0, 4 or 14 days before RNA and protein analyses. *Rest* expression was measured by RT-qPCR at (A) day 4, (B) day 0 and (C) day 14. (D) Western blot analysis showing REST level. *MeCP2* expression was measured by RT-qPCR at (E) day 4, (F) day 0 and (G) day 14. (H) Western blot analysis showing MeCP2 level. REST and MeCP2 were normalized to actin. The results are representative of 2 independent experiments performed in triplicate. Statistical analyses were performed using the Mann-Whitney test. *ns*, non-significant.
(TIF)

S1 Table Primer sequences. ApoE, Apolipoprotein E; Gapdh, Glyceraledhyde-3-phosphate dehydrogenase; Mecp2, methyl-CpG-binding protein2; REST, RE-1-silencing transcription factor; Sox2, (Sox determining region Y)-box2; TH, tyrosine hydroxylase.
(DOC)
