# Supplementary figures and images for: Borna Disease Virus Phosphoprotein Impairs the Developmental Program Controlling Neurogenesis and Reduces Human GABAergic Neurogenesis
Source: PLoS Pathog. 2015 Apr 29;11(4):e1004859. doi: 10.1371/journal.ppat.1004859 (PMC4414417; doi:10.1371/journal.ppat.1004859)

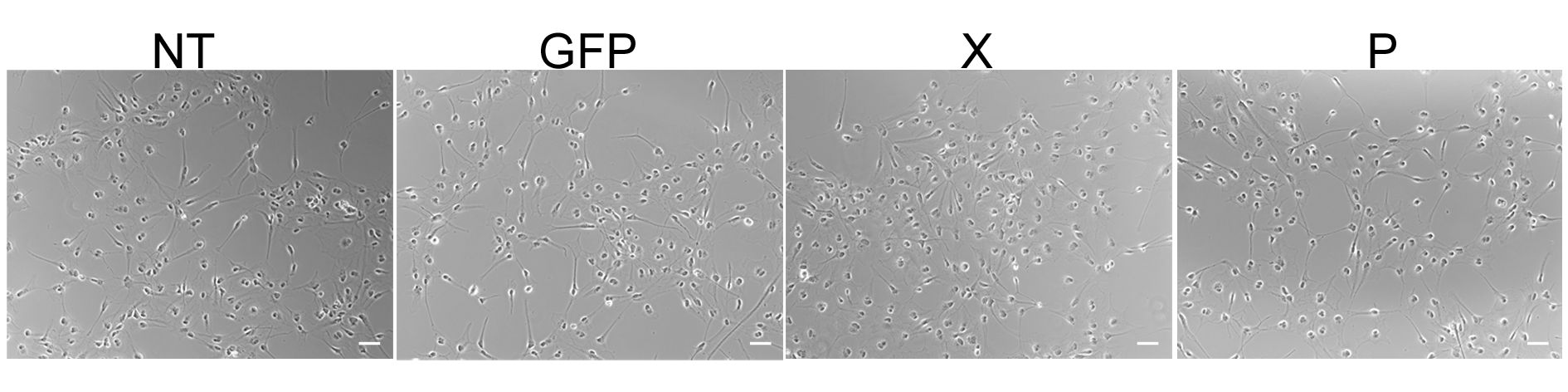

Supplement: S1 Fig — Representative phase-contrast photomicrographs of NT, gfp-, bdv-p- and bdv-x-expressing hNPCs at undifferentiated stage. Scale bar, 50 μm. (TIF) [file ppat.1004859.s001.tif]

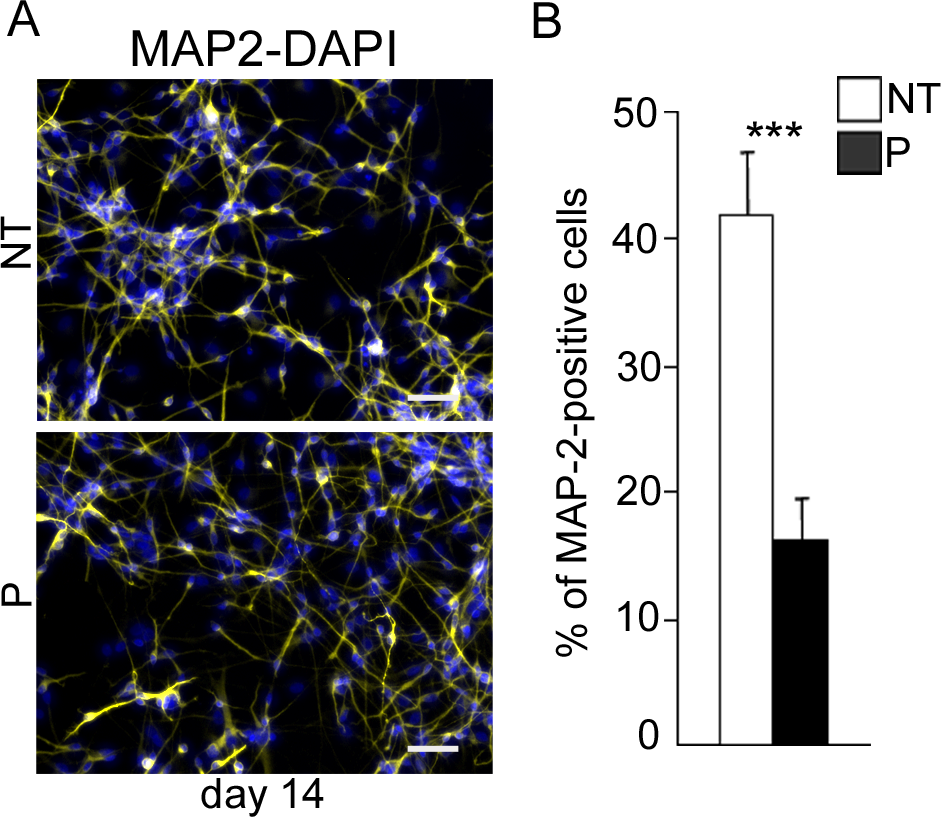

Supplement: S2 Fig — bdv-p-expressing hNPCs and their matched NT controls were induced to differentiate for 14 days and (A) immunostained with an antibody directed against MAP2, a neuronal marker (red). Nuclei were stained with DAPI (blue). (B) The percentage of neurons was determined based on enumeration of MAP2-positive cells. Results are representative of 2 independent experiments performed in triplicate. Statistical analysis was performed using the Mann-Whitney test. ***, p < 0.001, ns, non-significant (p > 0.5). Scale bar, 50 μm. (TIF) [file ppat.1004859.s002.tif]

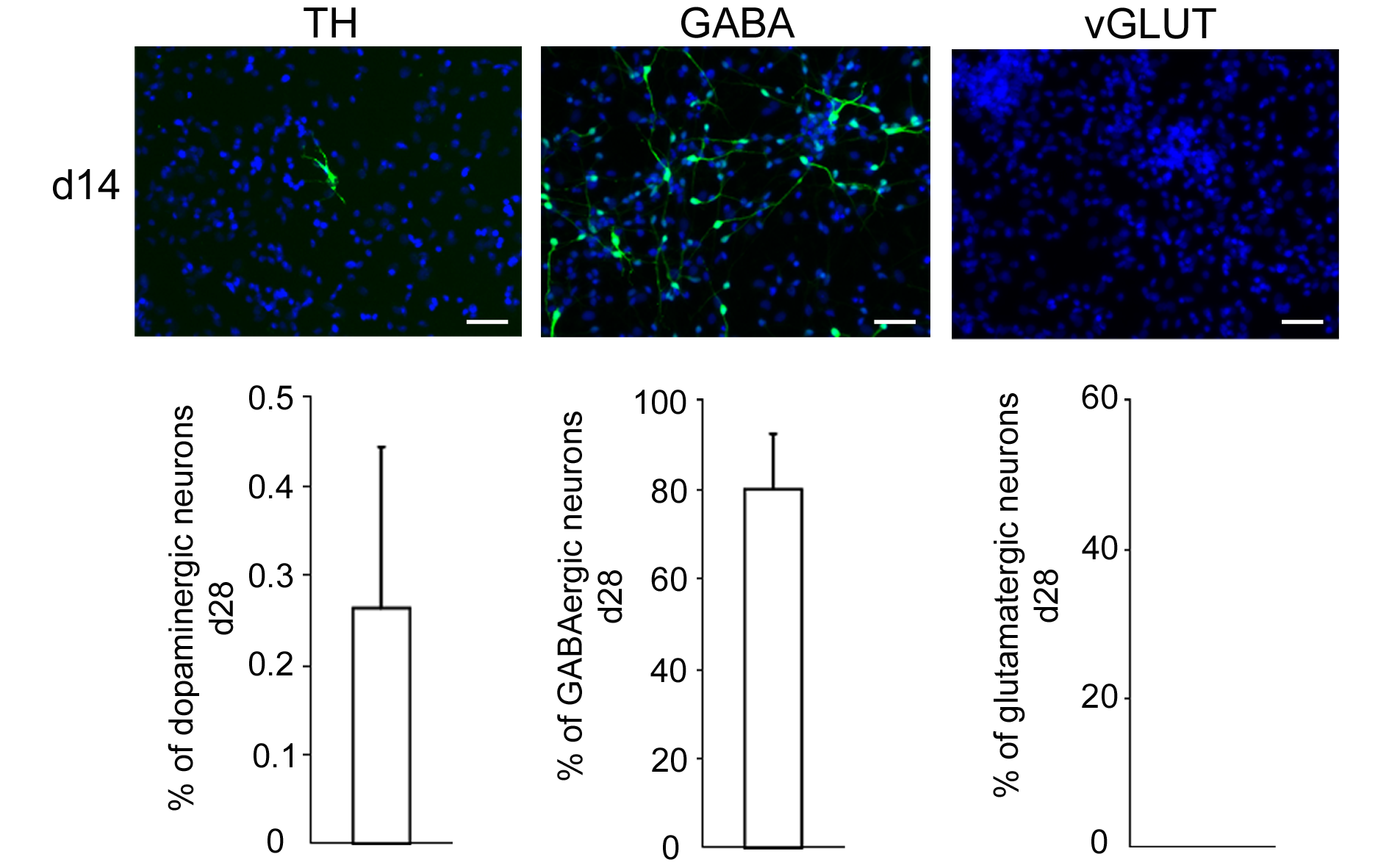

Supplement: S3 Fig — Non-transduced hNPCs were induced to differentiate for 28 days and (A) immunostained with antibodies directed against HuC/D (total neurons, red), GABA (GABAergic neurons, green), TH (dopaminergic neurons, green) and v-glut1/2 (glutamatergic neurons, green). Nuclei were counterstained with DAPI (blue). (B) Percentage of neuronal subtypes. Results are representative of 2 independent experiments performed in triplicate. Scale bar, 50 μm. (TIF) [file ppat.1004859.s003.tif]

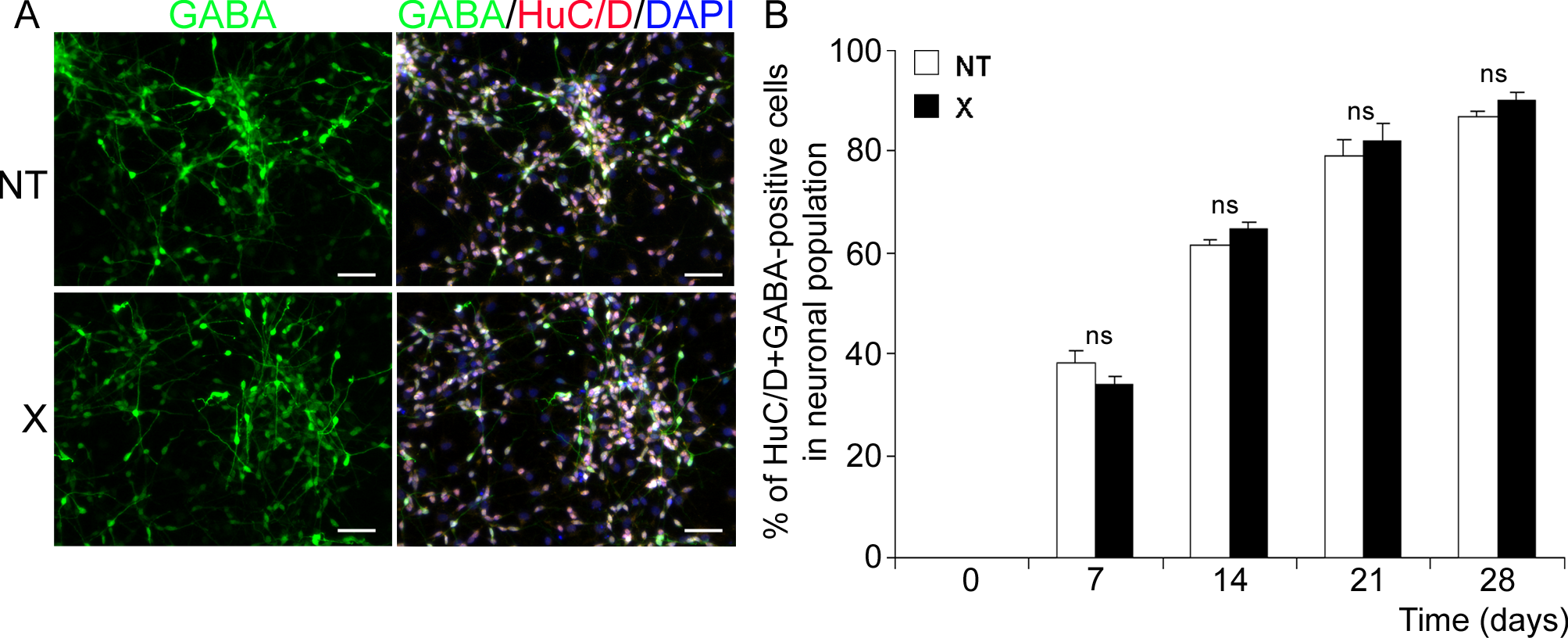

Supplement: S4 Fig — bdv-x-expressing hNPCs and their matched NT controls were induced to differentiate for 7, 14, 21 and 28 days and immunostained with antibodies directed against HuC/D (total neurons, red) and GABA (GABAergic neurons, green). (A) hNPCs differentiated for 14 days. Nuclei were counterstained with DAPI (blue). Scale bar, 50 μm. (B) Time-course analysis showing the percentage of GABAergic neurons in the total neuronal population. Results are representative of 2 independent experiments performed in triplicate. Statistical analyses were performed using the Mann-Whitney test. ***, p < 0.005, nd, non-determined. (TIF) [file ppat.1004859.s004.tif]

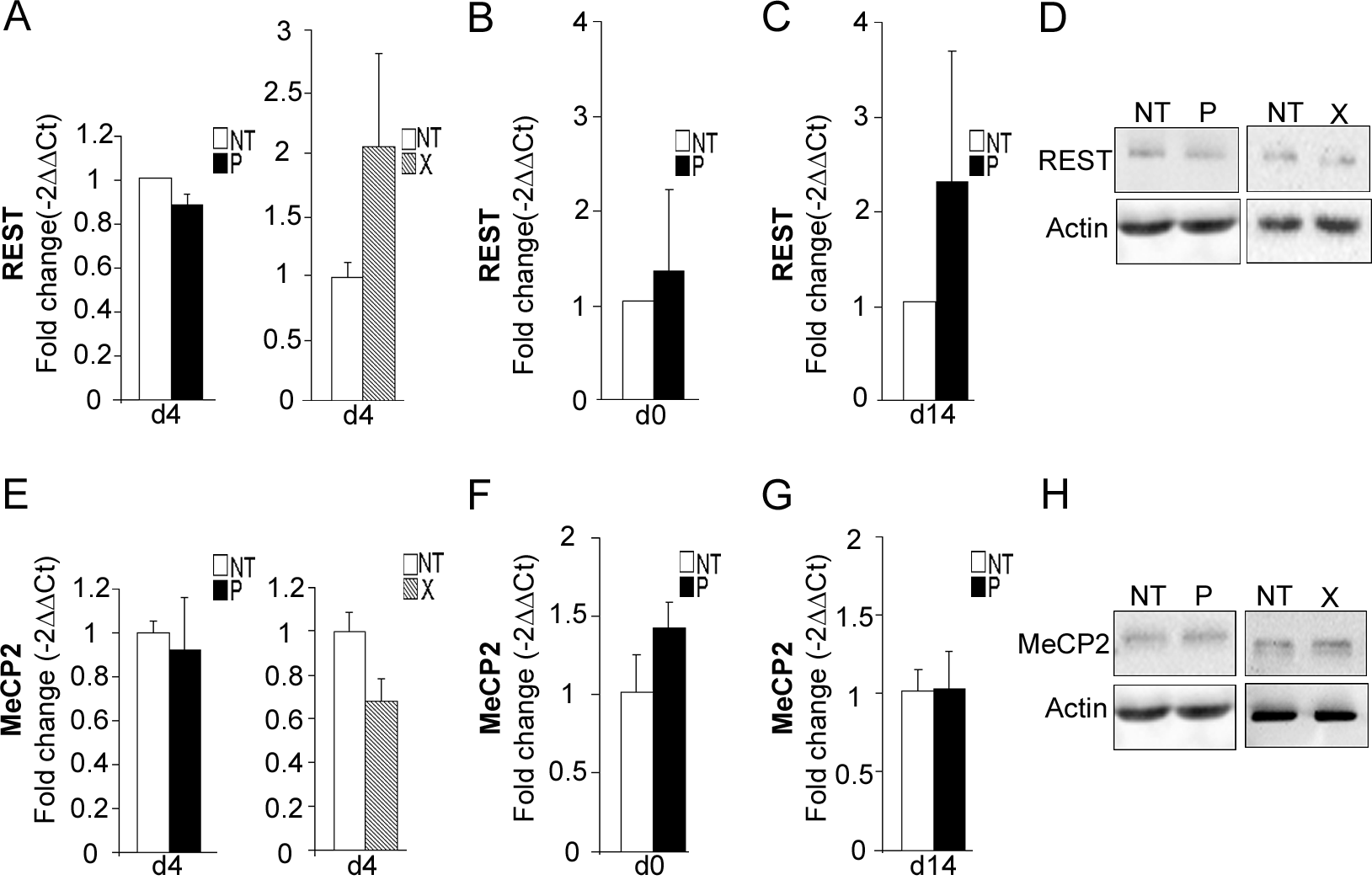

Supplement: S5 Fig — bdv-p- and bdv-x-expressing hNPCs and their matched NT controls were induced to differentiate for 0, 4 or 14 days before RNA and protein analyses. Rest expression was measured by RT-qPCR at (A) day 4, (B) day 0 and (C) day 14. (D) Western blot analysis showing REST level. MeCP2 expression was measured by RT-qPCR at (E) day 4, (F) day 0 and (G) day 14. (H) Western blot analysis showing MeCP2 level. REST and MeCP2 were normalized to actin. The results are representative of 2 independent experiments performed in triplicate. Statistical analyses were performed using the Mann-Whitney test. ns, non-significant. (TIF) [file ppat.1004859.s005.tif]
