# Supplementary material for: Borna Disease Virus Phosphoprotein Impairs the Developmental Program Controlling Neurogenesis and Reduces Human GABAergic Neurogenesis
Source: PLoS Pathog. 2015 Apr 29;11(4):e1004859. doi: 10.1371/journal.ppat.1004859 (PMC4414417; doi:10.1371/journal.ppat.1004859)
Supplement: S1 Table — ApoE, Apolipoprotein E; Gapdh, Glyceraledhyde-3-phosphate dehydrogenase; Mecp2, methyl-CpG-binding protein2; REST, RE-1-silencing transcription factor; Sox2, (Sox determining region Y)-box2; TH, tyrosine hydroxylase. (DOC) [file ppat.1004859.s006.doc]

| **gene** | **F/R** | **Sequences** |
| --- | --- | --- |
| ApoE | F | 5’-GGATCCTTGAGTCCTACTCAGC-3’ |
| ApoE | R | 5’-CAGCCCACAGAACCTTCATCT-3’ |
| Gapdh | F | 5’-CACCATCTTCCAGGAGCGAG-3’ |
| Gapdh | R | 5’-GAGATGATGACCCTTTTGGC-3’ |
| Mecp2 | F | 5’-GATCAATCCCCAGGGAAAAGC-3’ |
| Mecp2 | R | 5’ -CCTCTCCCAGTTACCGTGAAG-3’ |
| Nestin | F | 5’-CAGCGTTGGAACAGAGGTTGG-3’ |
| Nestin | R | 5’-TGGCACAGGTGTCTCAAGGGTAC-3’ |
| noggin | F | 5’ -CGAGCGAGATCAAAGGGCTA-3’ |
| noggin | R | 5’-CGACCACAGCCACATCTGT-3’ |
| Rest | F | 5’-GCAGCAAATGAGTCTCAGGA-3’ |
| Rest | R | 5’-ACCAAATGGCGATTGAGGTG-3’ |
| Scg10/Stathmin2 | F | 5’-TCCAGAAGAAACTGGAGGCT-3’ |
| Scg10/Stathmin2 | R | 5’-TCTCTGCCAATTGTTTCAGC-3’ |
| SOX2 | F | 5’-GGAAACTTTTGTCGGAGACG-3’ |
| SOX2 | R | 5’-CATGAGCGTCTTGGTTTTCC-3’ |
| TH | F | 5’-TTCTCTTACAAGGGGCCCAG-3’ |
| TH | R | 5’-CTTGTCATCCCTCCACCCAG-3’ |
